# Stretchable Complementary Split Ring Resonator (CSRR)-Based Radio Frequency (RF) Sensor for Strain Direction and Level Detection

**DOI:** 10.3390/s16101667

**Published:** 2016-10-11

**Authors:** Seunghyun Eom, Sungjoon Lim

**Affiliations:** School of Electrical and Electronics Engineering, College of Engineering, Chung-Ang University, 221 Heukseok-dong, Dongjak-gu, Seoul 156-756, Korea; umsh0303@gmail.com

**Keywords:** stretchable sensor, 3D printing, microfluidic channel, CSRR, EGaIn, Ecoflex

## Abstract

In this paper, we proposed a stretchable radio frequency (RF) sensor to detect strain direction and level. The stretchable sensor is composed of two complementary split ring resonators (CSRR) with microfluidic channels. In order to achieve stretchability, liquid metal (eutectic gallium-indium, EGaIn) and Ecoflex substrate are used. Microfluidic channels are built by Ecoflex elastomer and microfluidic channel frames. A three-dimensional (3D) printer is used for fabrication of microfluidic channel frames. Two CSRR resonators are designed to resonate 2.03 GHz and 3.68 GHz. When the proposed sensor is stretched from 0 to 8 mm along the +*x* direction, the resonant frequency is shifted from 3.68 GHz to 3.13 GHz. When the proposed sensor is stretched from 0 to 8 mm along the −*x* direction, the resonant frequency is shifted from 2.03 GHz to 1.78 GHz. Therefore, we can detect stretched length and direction from independent variation of two resonant frequencies.

## 1. Introduction

In order to realize stretchable features, depositing thin-film metals on elastomers [1,2,3,4,5,6,7], filling microfluidic channels with conducting materials [8,9] and Deep Reactive Ion Etching (DRIE) of silicon [10] are typically used. A common way to fabricate microfluidic channels is sealing a substrate (e.g., glass or PDMS (polydimethylsiloxane)) [11,12,13,14]. This is realized by special methods such as thermal evaporation, electro- deposition, e-beam evaporation, sputtering, patterning the metal (lithography), spin coating and ultraviolet exposure. Recently, microfluidic channels have been built by laser ablation [15,16] and a three-dimensional (3D) printer [17,18].

A complementary split ring resonator (CSRR) is a well-known structure. CSRR is the complementary structure of the split ring resonator (SRR). Because CSRR is usually etched on the ground plane of a substrate, it does not require additional area. Therefore, it has the benefit of size reduction.

**Figure 1:**
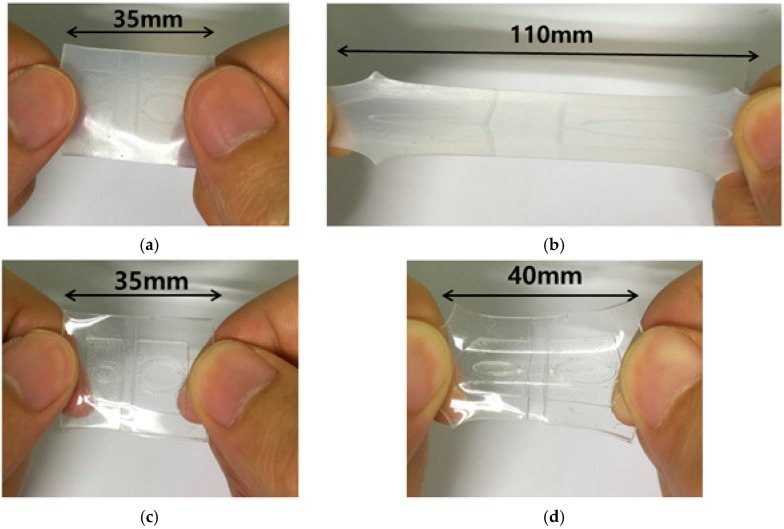
A comparison between Ecoflex and polydimethylsiloxane (PDMS) substrate: Ecoflex substrate at (**a**) non-stretched state and (**b**) stretched state. PDMS substrate at (**c**) non-stretched state and (**d**) stretched state.

Ecoflex 00-30 is skin-safe and has high tear strength (=38 pli) and high elongation at break (=900%), while PDMS-184 has tear strength (=15 pli) and elongation at break (=160%). Therefore, Ecoflex is suitable for stretchable applications. Figure 1 shows that an Ecoflex substrate with 1 mm thickness has higher stretchability than a PDMS substrate with 1 mm thickness. Recently, there have been many studies for stretchable applications using Ecoflex [19,20,21]. In case of flexible and stretchable applications, the filling of microfluidic channels with conductive material, liquid metal (eutectic gallium-indium, EGaIn, Ga 75.5% and In 24.5%) has been widely used because it is non-toxic and easy to inject into a microfluidic channel [22,23,24].

In this paper, a radio frequency (RF) complementary split ring resonator (CSRR) stretchable sensor is proposed in order to detect strain level. The proposed stretchable sensor can detect strain level from change of the resonant frequencies. Due to the existence of two different CSRRs, strain level along different directions can be independently detected. In order to simplify the fabrication process, a 3D printer is used for fabrication of microfluidic channels. High stretchable Ecoflex elastomer is used as dielectric material. Eutectic gallium-indium (EGaIn) liquid metal is used for conductive patterns.

## 2. Operating Principle and Design

**Figure 2:**
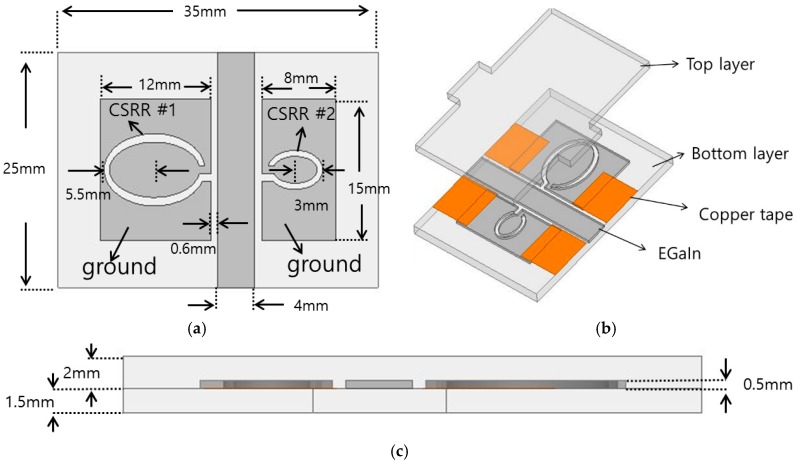
(**a**) Layout of the complementary split ring resonators (CSRRs); (**b**) Bird’s eye view of the proposed stretchable sensor; (**c**) Side view of the proposed stretchable sensor.

As illustrated in Figure 2a, the proposed RF stretchable sensor has two different complementary split ring resonators (CSRRs) using coplanar waveguide (CPW) technology. In order to realize stretchable conductive patterns, EGaIn is injected into the microfluidic channels. The thickness of conductive patterns is 0.5 mm. The boundary of EGaIn is assigned as finite conductivity with 3.4 × 106 Siemens/m. Microfluidic channels are built from two Ecoflex 00-30 (Smooth-On Inc., Easton, PA, USA) materials. Top and bottom Ecoflex layers have 1.5 mm and 2 mm thickness, respectively. The relative permittivity and dielectric loss of Ecoflex are 2.8 and 0.02, respectively. The bottom layer (thickness = 2 mm) has CPW (coplanar waveguide) channel (thickness = 0.5 mm) and two complementary split ring resonators (CSRRs) are different sizes.

**Figure 3:**
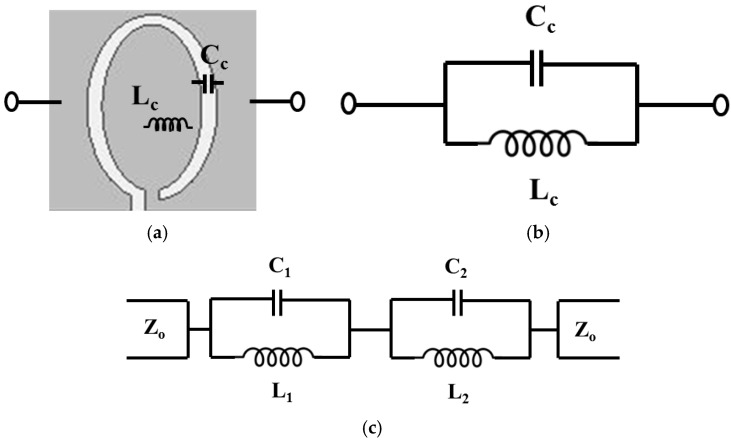
(**a**) Layout and (**b**) equivalent circuit model of the CSRR; (**c**) Equivalent circuit model of the proposed sensor with CSRR #1 and #2.

Two CSRRs are designed to resonate at 2.03 GHz and 3.63 GHz. Figure 3 shows the equivalent circuit model of the CSRR. The CSRR structure behaves as a parallel LC resonant circuit. The capacitance *C_c_* is the etched pattern of the CSRR ellipse. The inductance *L_c_* is the inner ellipse of the conductive pattern to the ground [25,26,27,28]. Therefore, the resonant frequency of the CSRR is determined by

(1) $$f=\frac{1}{2\pi \sqrt{L_{c}C_{c}}},$$

When one of two CSRRs is stretched without deforming the other CSRR, the electrical length of the stretched CSRR is increased which results in higher *L_c_*. Therefore, the resonant frequency of the stretched CSRR is decreased. In the meantime, the resonant frequency of the other CSRR is not changed because its electrical length is unchanged. Therefore, strain level from two CSRR can be independently detected from variation of resonant frequencies of two different CSRRs.

In order to design the proposed RF sensor, finite-element-method (FEM)-based ANSYS high frequency structure simulator (HFSS) is used. First of all, a 50-ohm CPW transmission line is designed as the feeding line. The width of the signal is 4 mm and the gap between signal and ground (GND) lines is 0.8 mm. Next, two CSRR are designed on the GND plane of the CPW. The CSRR #1 is realized on the left GND plane while the CSRR #2 is realized on the right GND plane. The resonant frequencies of the CSRR #1 and #2 are 2.03 GHz (*f*_1_) and 3.63 GHz (*f*_2_), respectively.

Figure 3c shows the equivalent circuit model of the proposed RF sensor with two CSRRs. Its circuit parameters (L_1_, C_1_, L_2_, and C_2_) are extracted from the S-parameters. The equivalent inductance (L_1_) and capacitance (C_1_) of the un-stretched CSRR #1 are 2.38 nH and 2.69 pF, respectively. The equivalent inductance (L_2_) and capacitance (C_2_) of the un-stretched CSRR #2 are 1.06 nH and 1.85 pF, respectively.

**Figure 4:**
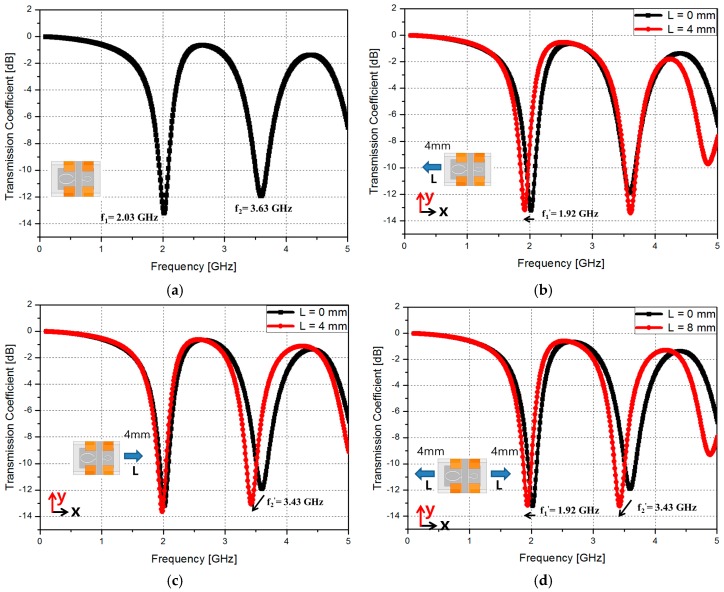
Simulated transmission coefficient results of the proposed stretchable sensor (**a**) when the sensor is not stretched; (**b**) when the proposed sensor is stretched by 4 mm along −*x* direction; (**c**) when the proposed sensor is stretched by 4 mm along +*x* direction and (**d**) when the proposed sensor is stretched by L = 4 mm along +*x* direction and L = 4 mm along −*x* direction.

The simulated transmission coefficients of the proposed RF sensor are shown in Figure 4. When the RF sensor is not stretched, the CSRRs #1 and #2 resonate at 2.03 GHz (*f*_1_) and 3.63 GHz (*f*_2_), respectively, as shown in Figure 4a.

Figure 4b shows the simulated transmission coefficient when the proposed sensor is stretched by L = 4 mm along −*x* direction and along +*x* direction is not stretched. The resonant frequency of *f*_1_ decreases from 2.03 to 1.92 GHz while *f*_2_ is not changed. Figure 4c shows the simulated transmission coefficient when the proposed sensor is stretched by L = 4 mm along +*x* direction and along −*x* direction is not stretched. The resonant frequency of *f*_2_ decreases from 3.63 to 3.43 GHz while *f*_1_ is not changed. Finally, when the proposed sensor is stretched by L = 4 mm along +*x* direction and L = 4 mm along −*x* direction, Figure 4d shows that f1 and f2 decrease from 2.03 to 1.92 GHz and from 3.63 to 3.43 GHz, respectively.

**Figure 5:**
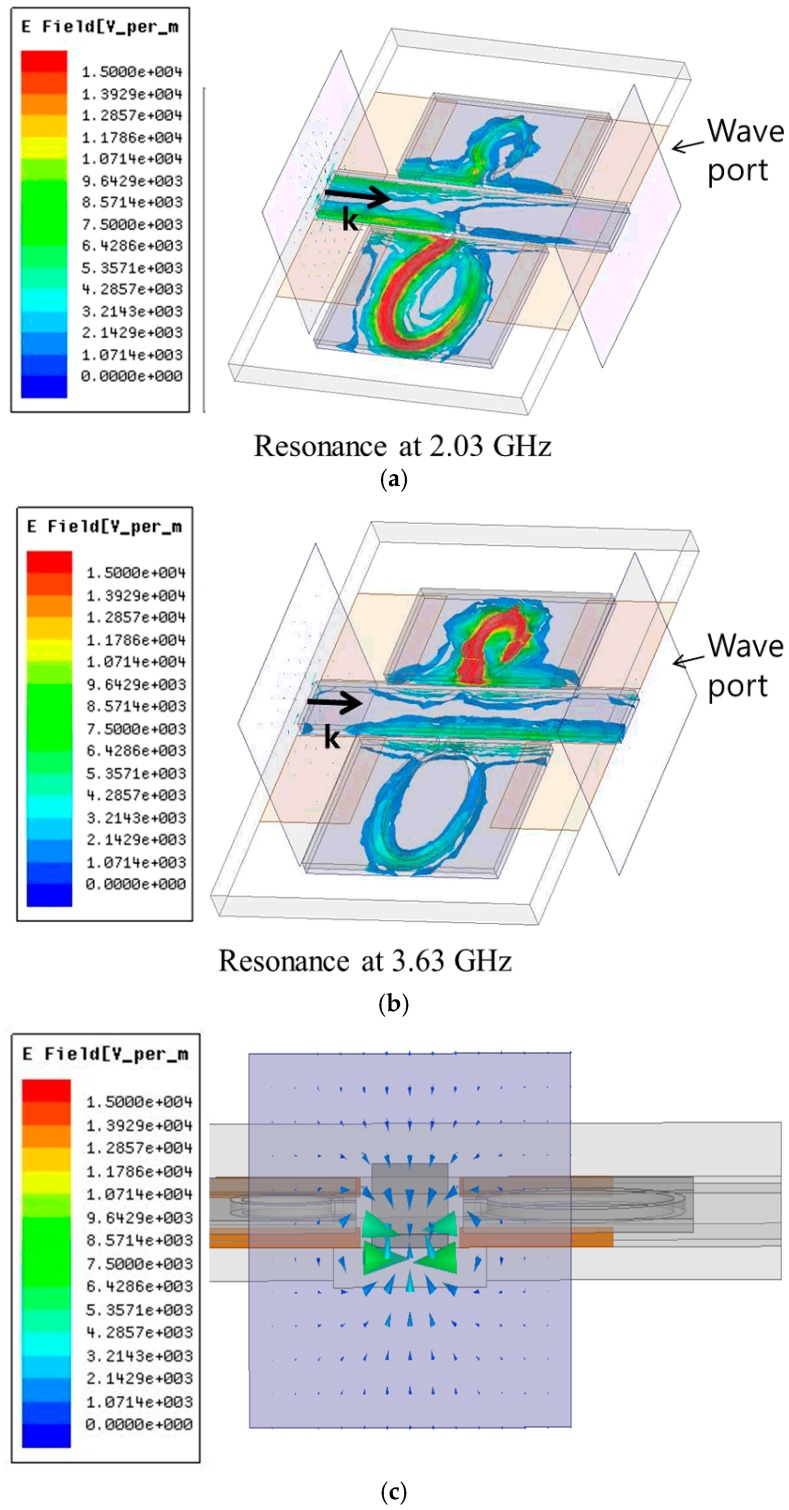
Simulated magnitude of electric field distribution of the proposed stretchable sensor at (**a**) 2.03 GHz and (**b**) 3.63 GHz; (**c**) Simulated vector of excited electric field on the wave port.

Figure 5 shows the magnitudes of electric field distribution when the proposed sensor is not stretched. The CSRR provides strong electric response at the resonant frequencies. At 2.03 GHz, electric resonance occurs from slot of the CSRR #1. At 3.63 GHz, electric resonant is generated from the slot of the CSRR #2.

## 3. Fabrications and Measurement Results

**Figure 6:**
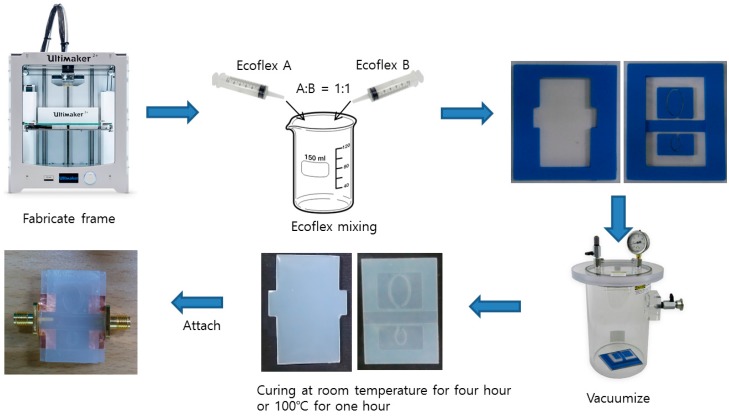
Fabrication process of the proposed stretchable sensor.

Figure 6 shows the fabrication process. The microfluidic channel frame is first built using a 3D printer (Ultimaker2, Ultimaker BV, Geldermalsen, Netherlands). After considering stretched liquid metal and printing resolution, the channel depth is 0.5 mm which becomes the thickness of liquid metal. Next, the substrate is realized using the Ecoflex. Ecoflex silicone elastomer base and curing agent is mixed at a ratio of 1:1. After mixing, a vacuum chamber is used to remove air bubbles of mixed solution. Although the mixed solution can be cured at a room temperature for four hours, it is cured at 100 °C for one hour in order to reduce a fabrication time. Before curing, we poured uncured solution to the microfluidic channel frame. A copper tape and SMA connector are attached to the cured substrate. Each layer is bonded by the uncured Ecoflex. Finally, in order to realize conductive patterns, EGaIn is injected into the microfluidic channels using the syringe.

**Figure 7:**
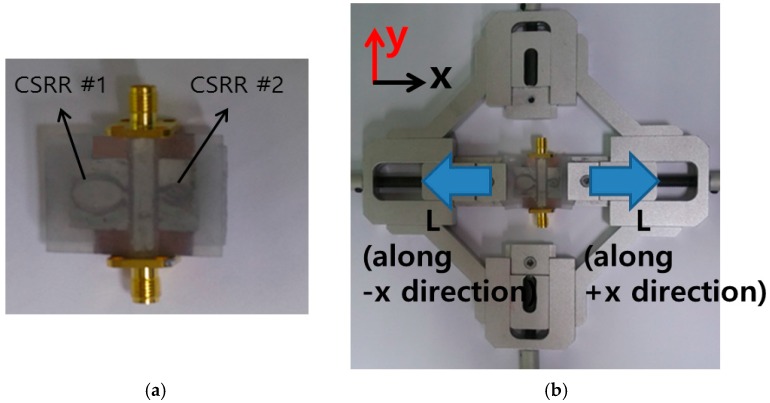
Pictures of (**a**) fabricated RF sensor and (**b**) the test setup. The fabricated sensor is fixed to a clamping fixture. The stretching length (L) can be manually varied from 0 mm to 16 mm (=8 mm along +*x* direction +8 mm along −*x* direction).

**Figure 8:**
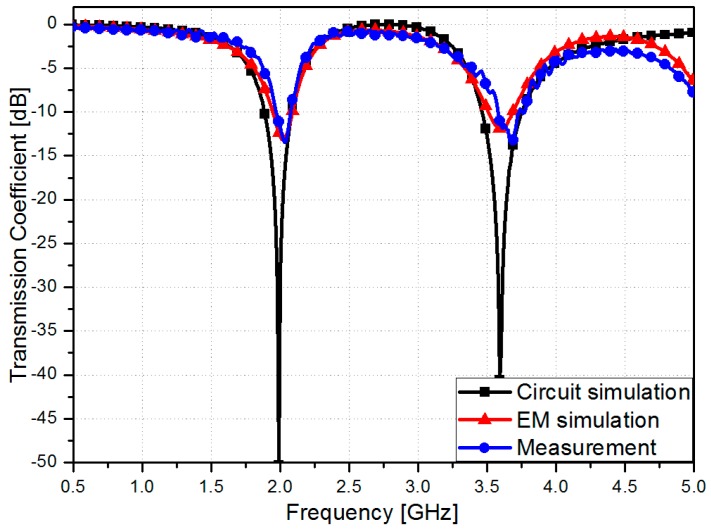
Transmission coefficients of the proposed RF sensor from circuit simulation, EM simulation and measurement.

Figure 7a shows the picture of the fabricated RF sensor prototype. The transmission coefficients of the fabricated RF sensor are measured using Anritsu MS2038C vector network analyzer. In order to stretch the fabricated RF sensor, the clamping fixture is used as shown in Figure 7b. In Figure 8, the simulated and measured transmission coefficients of the proposed RF sensor are compared when it is not stretched. The measured resonant frequencies are 2.03 GHz and 3.68 GHz while the simulated resonant frequencies are 2.03 GHz and 3.63 GHz. In EM simulation, the dielectric constant of Ecoflex is characterized at a low frequency around 2 GHz. Because the dielectric constant of Ecoflex is slightly decreased with a higher frequency, a slight difference is occurred at the resonant frequency of the CSRR #2.

**Figure 9:**
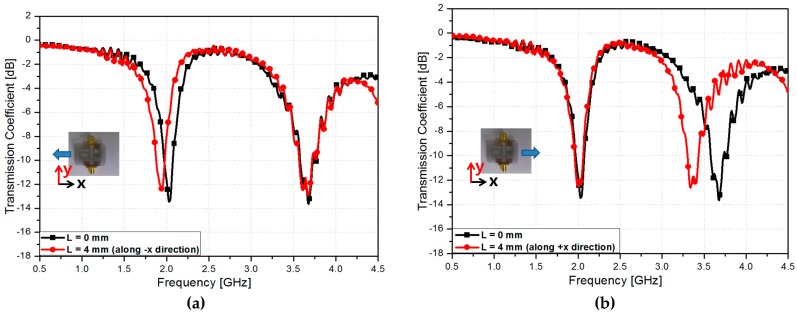

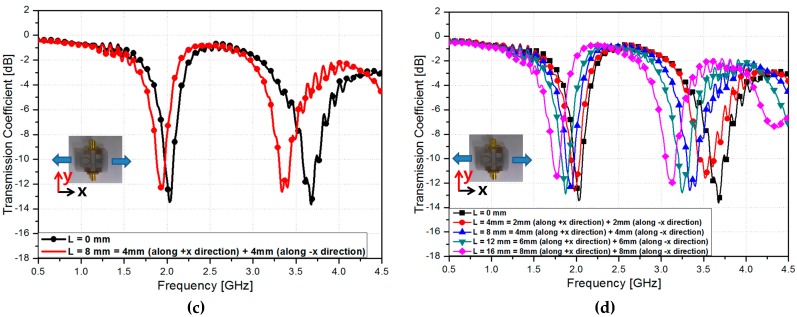
Measured transmission coefficients of the fabricated radio frequency (RF) sensor when (**a**) the fabricated sensor is stretched by L = 4 mm along −*x* direction; (**b**) the fabricated sensor is stretched by L = 4 mm along +*x* direction; (**c**) the fabricated sensor is stretched by L = 4 mm along +*x* direction and L = 4 mm along −*x* direction and (**d**) L is varied from 0 mm to 16 mm.

Figure 9 shows the measured transmission coefficients at different stretching length (L). Because the clamping fixture has a step of 0.2 mm, the minimum stretchable range is 0.57% stain. Due to limitation of the measurement setup, lower than 0.57% strain cannot be measured. Because of the mechanical strength of the Ecoflex material, the maximum detectable range is 22.86% of strain and critical frequencies are 1.78 GHz and 3.13 GHz which are the minimum and maximum frequencies, respectively. When the fabricated sensor is stretched by L = 4 mm along the −*x* direction and L = 0 mm along the +*x* direction, it is observed from Figure 9a that the resonant frequency *f*_1_ decreases from 2.03 to 1.97 GHz while *f*_2_ is not changed. When the fabricated sensor is stretched by L = 4 mm along +*x* direction and L = 0 mm along −*x* direction, it is observed from Figure 9b that the resonant frequency *f*_2_ decreases from 3.68 to 3.52 GHz while *f*_1_ is not changed. Figure 9c shows the measured transmission coefficients when the fabricated sensor is stretched by L = 4 mm along +*x* direction and L = 4 mm along −*x* direction. *f*_1_ and *f*_2_ decrease from 2.03 to 1.97 GHz and from 3.68 to 3.52 GHz, respectively. When the sensor is not stretched, the measured Q-factors of CSRR #1 and #2 are 4.8333 and 4.58, respectively. When the stretching length (L) is simultaneously varied along the +*x* and −*x* directions, the transmission coefficients are measured as shown in Figure 9d. L is varied from 0 to 16 mm in increments of 4 mm (L of +*x* direction is varied from 0 to 8 mm in increments of 2 mm and L of −*x* direction is varied from 0 to 8 mm in increments of 2 mm). Their resonant frequencies are listed in Table 1. Although the stretched physical lengths along +*x* and −*x* directions are same, the frequency shift is different because the electrical length at the resonant frequencies of the CSRR #1 and #2 are different each other.

The frequency change (*Δf*) is defined as

(2) $$\Delta f=\left|f_{non-stretched}-f_{stretched}\right|,$$

where *f_non-stretched_* and *f_stretched_* are the measured resonant frequencies in the non-stretched and stretched states, respectively. Let us now define relative resonant frequency change as

(3) $$\mathrm{Relative}\;\mathrm{frequency}\;\mathrm{change}\;=\;\frac{\Delta f}{f_{0}}$$

where *f*_0_ is the reference frequency at non-stretched state.

In this work, the sensitivity (*S_L_*) is defined as

(4) $$S_{L}=\frac{\delta \left(\Delta f/f_{0}\right)}{\delta l}\;\left({\mathrm{mm}}^{-1}\right)$$

where L is stretching length.

**Figure 10:**
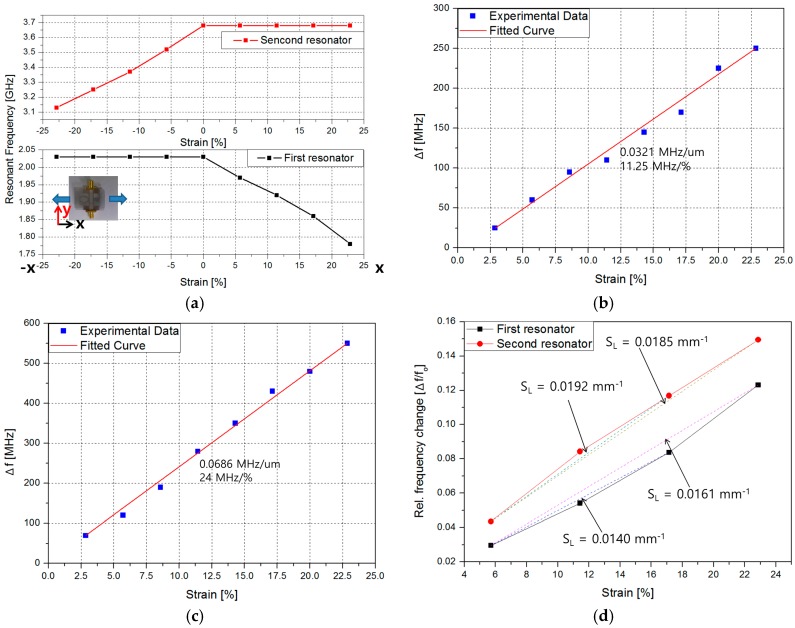
Experimental characterization of the proposed sensor under tension: (**a**) resonant frequency versus stretching length along +*x* and −*x* direction; (**b**) frequency change (*Δf*) when only CSRR #1 is stretched from 2.86% to 22.86%; and (**c**) frequency change (*Δf*) when only CSRR #2 is stretched from 2.86% to 22.86%; and (**d**) relative frequency change (*Δf*/*f*_0_) of CSRR #1 and #2.

Figure 10a shows the measured resonant frequencies when the fabricated sensor is stretched along +*x* and −*x* direction from −22.86% to +22.86%. When L is increased from 0% to 22.86% along +*x* direction, the resonant frequency is decreased from 2.03 to 1.78 GHz. When L is increased from 0% to 22.86% along −*x* direction, the resonant frequency is decreased from 3.63 to 3.13 GHz. Figure 10b,c show the frequency change (*Δf*) versus stretching length for CSRR #1 and #2, respectively. The relationship is linear and their fitted curves are *y* = 11.25*x* − 7.175 (MHz/%) and *y* = 24*x* + 1.36 (MHz/%), respectively. Figure 10d shows the relative frequency change (*Δf*/*f*_0_) versus strain level from 5.72% to 22.86% for each CSRR. The sensitivity is also indicated as maximum 0.0192 mm^−1^.

In Table 2, the proposed sensor is compared with other RF strain sensors in [29,30,31,32,33]. Because the proposed sensor is built on liquid metal and Ecoflex substrate, wider frequency tuning range and higher strain level are achieved compared with other sensors. In addition, rapid prototyping with 3D printing provides the flexibility required to make this crucial trial and error process possible for physical products. Compared with RF sensors with low lossy substrates in [32,33], the Q-factor of the proposed sensor is relatively low due to dielectric loss of stretchable materials.

## 4. Conclusions

In conclusion, we proposed a stretchable RF sensor. The proposed sensor consists of the Ecoflex layers with microfluidic channel. Each layer is bonded by uncured Ecoflex. 3D-printing technology is adopted to realize the conductive CSRR patterns with liquid metal on stretchable Ecoflex substrate. The microfluidic channel frames with Ecoflex elastomer is used for the stretchable substrate. The proposed stretchable sensor can detect the resonant frequency change. When the fabricated sensor is stretched along the +*x* and −*x* direction from −22.86% to +22.86%, the resonant frequencies of CSRR#1 and CSRR #2 are changed from 2.03 GHz to 1.78 GHz and from 3.68 GHz to 3.13 GHz, respectively. The relationship of frequency change (*Δf*) versus stretching length for CSRR #1 and #2 is linear. The fitted curves of CSRR #1 and CSRR #2 are *y* = 11.25*x* − 7.175 and *y* = 24*x* + 1.36. When we stretched the proposed stretchable sensor, the resonant frequency is changed because the electrical length of resonator is changed. Because each resonator is working independently, we can detect direction and stretched length depending on strain level from independent variation of two resonant frequencies. The Q-factor of the proposed sensor can be further improved by using low lossy stretchable materials.

## Figures and Tables

**Table 1:** Relationship between the resonant frequency and strain level of L.

Strain (%)	CSRR #1 (GHz)	CSRR #2 (GHz)
0	2.03	3.68
11.42 = 5.71 (along +*x* direction) + 5.71 (along −*x* direction)	1.97	3.52
22.84 = 11.42 (along +*x* direction) + 11.42 (along −*x* direction)	1.92	3.37
34.28 = 17.14 (along +*x* direction) + 17.14 (along −*x* direction)	1.86	3.25
45.72 = 22.86 (along +*x* direction) + 22.86 (along −*x* direction)	1.78	3.13

**Table 2:** Comparison Table of Proposed Sensor and Other RF Strain Sensors.

	This Work	[29]	[30]	[31]	[32]	[33]
Substrate	Ecoflex	Kapton tape	Si	PDMS	RT/Duroid 5880	Kapton
Conductive Material	EgaIn	Au	Au	Au	Cu	Cu/Al
Fabrication Method	3D printing	Lithography, Evaporation	Lithography, Evaporation	Spin coating, Evaporation, Photolithography	Lithography	Lithography, Manual assembly
Maximum Strain Level (%)	45.72	N/A	N/A	9.78	0.2	1
Tuning Ratio (%)	12.31, 14.95	5.69	0.21	5.14	0.14	2.35
Number Of Detect Direction	2	1	1	2	1	2
Resonant Frequency (GHz)	2.03, 3.68	12.3	0.4742	700	5	3.62
Q factor	4.58~4.83	12–18 ^†^	N/A	3~4 ^†^	40~49 ^†^	40~50 ^†^
Resonator	Metamaterial	Metamaterial	Metamaterial	Metamaterial	Patch Antenna	Open loop

^†^ It is estimated from frequency response figures.
